# Co-infection of intestinal helminths in humans and animals in the Philippines

**DOI:** 10.1093/trstmh/trac002

**Published:** 2022-02-16

**Authors:** Olumayowa T Kajero, Eva Janoušková, Emmanuel A Bakare, Vicente Belizario, Billy Divina, Allen Jethro Alonte, Sheina Macy Manalo, Vachel Gay Paller, Martha Betson, Joaquin M Prada

**Affiliations:** School of Veterinary Medicine, Faculty of Health & Medical Sciences, University of Surrey, GU2 7AL, UK; School of Veterinary Medicine, Faculty of Health & Medical Sciences, University of Surrey, GU2 7AL, UK; Biomathematics and Applied Mathematical Modelling Research Group, Modelling Simulation and Data Science Network, Department of Mathematics, Federal University Oye-Ekiti, 371104, Ekiti, Nigeria; College of Public Health, University of Philippines, Manila, 1000, Philippines; Department of Veterinary Paraclinical Sciences, College of Veterinary Medicine, University of the Philippines Los Baños, 4030, Philippines; Animal Biology Division, Institute of Biological Sciences, University of the Philippines, Los Baños, 4030, Philippines; Department of Veterinary Paraclinical Sciences, College of Veterinary Medicine, University of the Philippines Los Baños, 4030, Philippines; Department of Veterinary Paraclinical Sciences, College of Veterinary Medicine, University of the Philippines Los Baños, 4030, Philippines; School of Veterinary Medicine, Faculty of Health & Medical Sciences, University of Surrey, GU2 7AL, UK; School of Veterinary Medicine, Faculty of Health & Medical Sciences, University of Surrey, GU2 7AL, UK

**Keywords:** co-infection, epidemiology, Philippines, strongyle, Toxocara, Helminths

## Abstract

**Background:**

A large number of studies have assessed risk factors for infection with soil-transmitted helminths (STH), but few have investigated the interactions between the different parasites or compared these between host species across hosts. Here, we assessed the associations between *Ascaris, Trichuris*, hookworm, strongyle and *Toxocara* infections in the Philippines in human and animal hosts.

**Methods:**

Faecal samples were collected from humans and animals (dogs, cats and pigs) in 252 households from four villages in southern Philippines and intestinal helminth infections were assessed by microscopy. Associations between worm species were assessed using multiple logistic regression.

**Results:**

*Ascaris* infections showed a similar prevalence in humans (13.9%) and pigs (13.7%). Hookworm was the most prevalent infection in dogs (48%); the most prevalent infection in pigs was strongyles (42%). The prevalences of hookworm and *Toxocara* in cats were similar (41%). Statistically significant associations were observed between *Ascaris* and *Trichuris* and between *Ascaris* and hookworm infections in humans, and also between *Ascaris* and *Trichuris* infections in pigs. Dual and triple infections were observed, which were more common in dogs, cats and pigs than in humans.

**Conclusions:**

Associations are likely to exist between STH species in humans and animals, possibly due to shared exposures and transmission routes. Individual factors and behaviours will play a key role in the occurrence of co-infections, which will have effects on disease severity. Moreover, the implications of co-infection for the emergence of zoonoses need to be explored further.

## Introduction

More than 1.5 billion people, or 24% of the world's population, are reported by the WHO to be infected with soil-transmitted helminths (STHs) globally.^1^ These infections cause significant morbidity in >450 million people, resulting in >39 million disability-adjusted life years lost worldwide.^2^ Intestinal helminth infections are also ubiquitous in livestock and companion animals. Transmission is mainly through a faecal-oral route involving ingestion of parasite eggs, due to the consumption of drinking water contaminated with human and animal faeces, the consumption of crops which have been fertilized using manure, poor personal hygiene and proximity to infected animals. Alternatively, the eggs can hatch in the soil and develop to an infective larval stage that can penetrate the skin directly (hookworm).^2^ In infected individuals, the eggs or larvae develop into adult worms that produce eggs and complete the lifecycle.^3^ The relatively high prevalence of intestinal helminth infections in dogs and pigs, and the large animal populations worldwide, could facilitate zoonotic transmission.^4^

There are >200 known types of zoonoses^5^ and around 60% of the 300 infectious agents identified during 1940–2004 were classified as zoonoses.^6^ Zoonotic transmission of intestinal helminths in southeast Asia, with consideration of their hosts, transmission, clinical presentation, geographical distribution and control measures, were recently reviewed by Betson et al.^2^

There is widespread distribution of helminth infections in humans in tropical and subtropical areas, with the greatest numbers occurring in sub-Saharan Africa, the Americas and Asia.^1^ The risk groups include preschool-age and school-age children, women of reproductive age (including pregnant women in their second and third trimesters and breastfeeding women) and adults in certain high-risk occupations such as tea-pickers or miners.^1^ More than 267 million preschool-age children and >568 million school-age children live in areas where these parasites are intensively transmitted, requiring treatment and preventive interventions.^1^ STHs pose a significant health problem in the Philippines, where it was reported that the prevalence of STH infections among school-age children was about 74%.^7^

Co-infections with multiple parasites, where the infectious agents coexist in the same host, have been mainly studied in humans, with a prevalence as high as 80% found in some communities.^8,9^ In highly endemic settings, nearly every new incident infection with helminths is likely to constitute some sort of co-infection. These co-infections may arise from many different external factors, such as shared environments and infection routes, and be facilitated or hampered by direct or indirect interactions between parasite species. However, the nature of these interactions inside the host remain unknown;^10^ some may be synergistic, where the presence of one parasite may lead to subsequent infections by other parasites; or alternatively they may be antagonistic, where competition may occur between parasites sharing a similar ecological niche in the host. An analysis conducted by Lepper and colleagues,^11^ using a cross-sectional study of STH infections in Sri Lanka, reported positive associations between *Trichuris trichiura* and both *Necator americanus* and *Ascaris lumbricoides*, but not between *N. americanus* and *Ascaris.*

Studies looking at co-infections are crucial in epidemiology, particularly as enhancing disease prevention measures would lead to reduction in exposure to multiple parasites.^10^

Our aim here is to investigate how these co-infections occur across a number of hosts, and whether the same parasite species appear recurrently in co-infections. In this study, we examine cross-sectional data from four rural villages in the Philippines, through an investigation of the epidemiology of intestinal helminths circulating in humans and animals (dogs, cats and pigs) living in the same household. The main species of STHs that infect humans, and thus the focus here, are *Ascaris* (roundworm), *Trichuris* (whipworm) and hookworm, but we also consider major infections affecting animal hosts, strongyles and *Toxocara*, with an emphasis on potential associations in prevalence between the worm species. Furthermore, this study provides a unique opportunity to gain better fundamental knowledge on infectious agent interactions and to investigate the role and consequences of co-infections in the emergence of zoonotic transmission,^9^ which could help inform interventions across hosts. Indeed, the presence of animals in close contact with humans could play a large role in disease persistence.

## Materials and Methods

### Study setting and design

Data collection was conducted as part of the Zoonotic Transmission of Intestinal Parasites (ZooTRIP) project^12^ and was carried out in four villages (Bunawan, Trento, Mainit and San Isidro), in the provinces of Agusan del Sur and Surigao del Norte, Mindanao, the Philippines. The collection of samples for the data took place in October and November 2019. Villages were selected based on known endemicity of intestinal helminths, the willingness of local government units to cooperate, accessibility of communities and the security situation.

Recruitment to the ZooTRIP project^12^ was conducted at household level. Households were selected at random from a list within each village and household members aged 10–60 y were invited to participate in the study. Information sheets tailored to different age groups in the local dialect were provided to study participants and explained to them by trained field team members. Written informed consent was obtained from adults and from guardians of participants aged <18 y. Written assent was obtained from children aged 12–17 y and verbal assent from children aged 10–11 y. If participants were illiterate, they were asked to document consent by means of a thumbprint in the presence of a literate witness, who signed the form.

Participants were provided with a stool collection kit containing a specimen cup, spatula and faecal sample collection instructions prior to the scheduled day of collection. An appointment was then made for participants to provide the stool sample and take part in the study at a convenient central location in the village. For school children participating in the study, sample and data collection took place in their respective schools.

A questionnaire was conducted for each participating household, where respondents provided relevant demographic information, such as gender, age and occupation, as well as knowledge, attitude and perception towards intestinal helminths and exposure information, such as data on the presence and characteristics of domestic animals, animal husbandry practices, hygiene, sanitation and toilet facilities.

Faecal samples were also collected after voiding from dogs, cats and pigs associated with households involved in the study. If faecal samples were not present, labelled stool containers were left to the animal owners and samples were then collected by Barangay health workers the next day. If any of them were not fresh, they would only be 1–3 d old. The consistency of stool samples was checked to ensure that samples were not too old to interfere with the diagnosis; 95% ethanol was immediately added to samples to preserve them. The global positioning system coordinates of participating households and sampling locations were recorded.

A total of 252 households were enrolled in the study and faecal samples were obtained from 300 adults, 385 school-age children, 91 dogs, 27 cats and 136 pigs.

### Diagnosis of helminth infections

Faecal samples collected from humans were transported to the field laboratory in a cold chain, processed using the Kato–Katz technique and examined to determine the presence of intestinal helminths. Two aliquots were prepared from each sample, and the number of eggs was determined in each aliquot. The average egg count was computed, and the number of eggs per gram of faeces was calculated. As part of quality control, all positive slides and 10% of the negative slides were re-examined by a reference microscopist. Discrepancies between readings were resolved as described.^13^ Animal faecal samples were processed and examined using flotation with Sheather's solution and sedimentation techniques described by Zajac and Conboy.^14^ Egg parasites were identified based on morphological features and size described by Zajac and Conboy.^14^

### Data analysis

The probability of infection with each parasite and the effect of other parasites species was modelled using multiple logistic regressions, which have been used to assess associations between intestinal worm species.^11^ It is important to note that we refer to association in terms of co-occurrence frequency, rather than biological interaction. The ORs, as well as the p-values, were calculated for the predictor variables, and a 0.05 significance threshold was applied. All models considered are shown in Table 1. The McFadden's R-squared was calculated for each model to assess the amount of variation in the data explained. In these models, faecal worm egg counts were transformed to a binary response variable, with 0 for all negatives (i.e. zero counts) and 1 for all positive (non-zero) counts of eggs.

**Table 1.** Summary of host and parasite species considered in the logistic regression models

Host	Species (binary response variables)	Predictor variables
**Humans**	*Ascaris*	Location
		Age
		*Trichuris*
		Hookworm
	*Trichuris*	Location
		Age
		*Ascaris*
		Hookworm
	Hookworm	Location
		Age
		*Ascaris*
		*Trichuris*
**Dogs and cats**	*Trichuris*	Location
		Hookworm
		*Toxocara*
	Hookworm	Location
		*Trichuris*
		*Toxocara*
	*Toxocara*	Location
		Hookworm
		*Trichuris*
**Pigs**	*Ascaris*	Location
		Strongyle
		*Trichuris*
	Strongyle	Location
		*Ascaris*
		*Trichuris*
	*Trichuris*	Location
		*Ascaris*
		Strongyle

For each intestinal helminth species considered in this study for a particular host, a model considering the presence of this species as an output variable was used, while including the presence of other relevant parasites as predictor variables. Other predictors included were location (village) as a fixed effect, and age when considering human hosts (to study the relevance of age in co-infection); gender (in humans) was originally considered, but it was removed as it had no effect in any of the models. As location is used as a fixed effect, the results are referenced to the first category, Bunawan. Households were considered as a random effect, to control for the fact that we have more than one individual per household. The combinations of hosts and parasites considered are summarised in Table 1.

We further assessed whether co-infection was random (i.e. independent of parasite species). First, we took the observed prevalences as the probabilities of being infected with each helminth species and calculated the CIs. Subsequently, the expected co-infection prevalences were calculated as the proportions that would be expected if the species were distributed randomly with respect to each other in the population (i.e. multiplying the probabilities of each considered event, infected or not infected, for each infection). For example, the expected probability of a triple co-infection in humans was calculated by multiplying the observed prevalences of all three infections considered in humans, *Ascaris, Trichuris* and hookworm.

Statistical analysis was carried out using R version 1.2.5033^15^ and RStudio^16^ using the glmer function from the lme4 package^17^ to compute the generalised linear mixed models.

## Results

**Figure 1.**
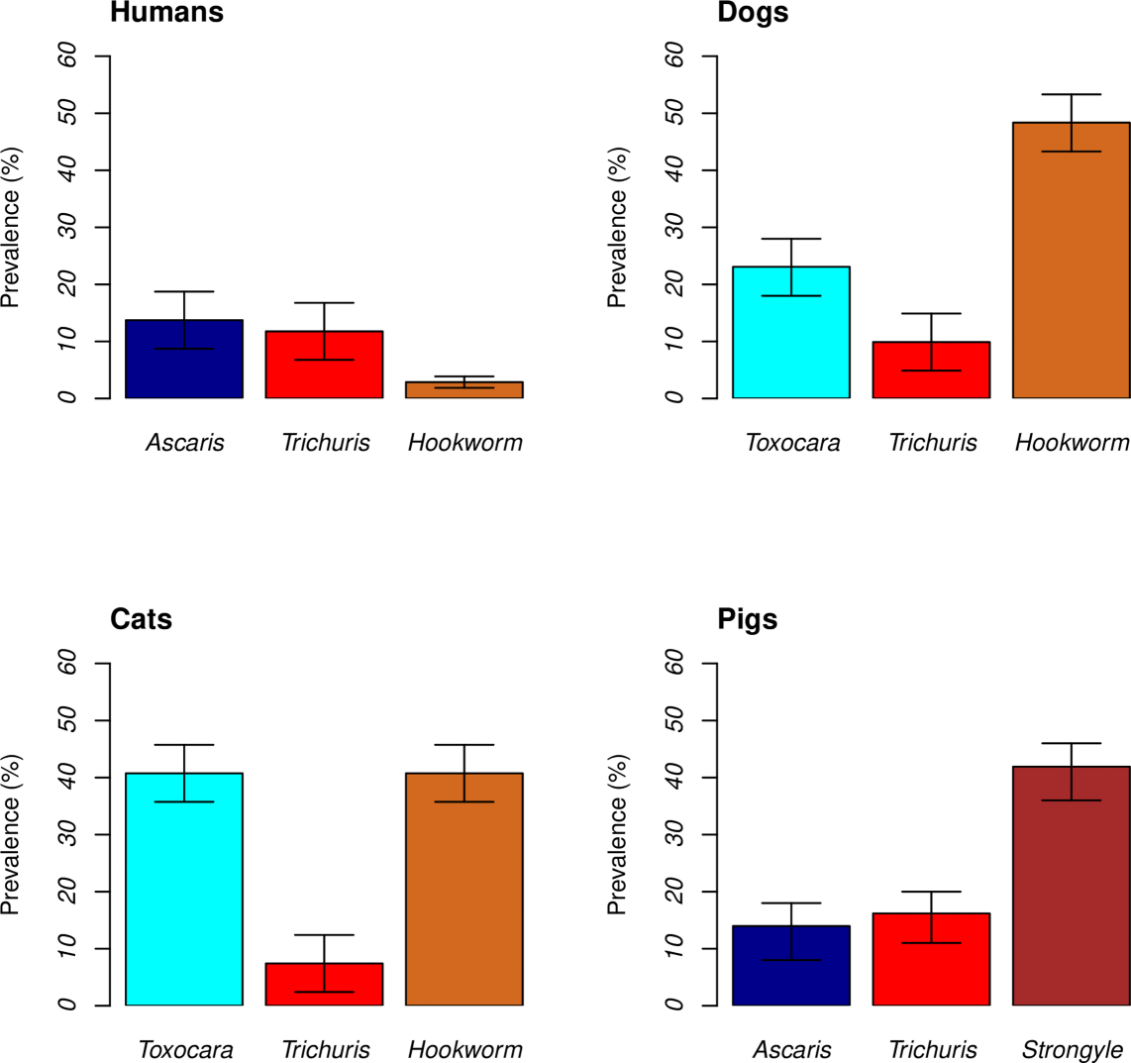
Prevalence of intestinal helminth infections in humans, dogs, cats and pigs. The total numbers of humans, dogs, cats and pigs sampled were 663, 91, 27 and 136, respectively.

The highest parasite prevalences were found in animals, with hookworm infection prevalences of 41% (11/27; 95% CI 36 to 46%) in cats and 48% (44/91; 95% CI 43 to 54%) in dogs, *Toxocara* prevalences of 41% (11/27; 95% CI 36 to 46%) in cats and strongyle infection prevalences of 42% (57/136; 95% CI 36 to 47%) in pigs. Human samples revealed comparatively lower parasite prevalences, ranging from 2% (95% CI 0.95 to 2.8%) to 13.9% (95% CI 9 to 18%), 91/663 positive for *Ascaris*, 78/663 for *Trichuris* and 19/663 for hookworm (Figure 1).

The ORs, p-values and McFadden's R-squared values calculated across all four host species are shown in Table 2. An analysis excluding age in humans is shown in Table S1.

**Table 2.** Multiple logistic regressions identifying which factors affect the presence of infections (species variables). Household is considered as a random effect to account for individuals in the same household, while the other variables are included as fixed effects. Age is included only for humans. Asterisks in the p-values denote statistically significant results

Host	Species	Variable	OR (95% CI)	p-value	McFadden's R^2^
**Humans**	*Ascaris*	*Trichuris* Hookworm Age Location Mainit Location San Isidro Location Trento	3.81 (1.96 to 7.39) 3.66 (1.02 to 13.01) 0.98 (0.96 to 1.00) 4.27 (1.56 to 11.68) 2.17 (0.74 to 6.31) 0.59 (0.17 to 2.06)	0.0002*** NS 0.0356* 0.0046** NS NS	0.09
	*Trichuris*	*Ascaris* Hookworm Age Location Mainit Location San Isidro Location Trento	4.15 (2.10 to 8.17) 1.19 (0.30 to 4.83) 0.98 (0.96 to 1.00) 10.97 (2.35 to 51.33) 34.99 (7.51 to 163.11) 2.22 (0.36 to 13.70)	3.9×10^–5^*** NS NS 0.0023** 6.0×10^–6^ *** NS	0.17
	Hookworm	*Ascaris* *Trichuris* Age Location Mainit Location San Isidro Location Trento	3.51 (1.13 to 10.91) 1.11 (0.33 to 3.72) 1.03 (1.00 to 1.06) 2.94×10^–8^ (0.00) 5.80 (1.23 to 27.38) 1.86 (0.30 to 11.37)	0.03* NS NS NS 0.03* NS	0.19
**Dogs**	*Trichuris*	Hookworm *Toxocara* Location Mainit Location San Isidro Location Trento	3.39×10^–1^ (2.58×10^–4^ to 4.46×10^2^) 7.98×10^–5^ (2.34×10^–5^ to 2.72) 2.47 (6.11×10^–4^ to 9.94×10^3^) 1.43 (2.72×10^–5^ to 7.50×10^4^) 2.01 (1.73×10^–4^ to 2.34×10^4^)	NS NS NS NS NS	0.30
	Hookworm	*Trichuris* *Toxocara* Location Mainit Location San Isidro Location Trento	0.56 (0.13 to 2.50) 2.19 (0.74 to 6.49) 0.80 (0.27 to 2.42) 0.25 (0.04 to 1.45) 1.23 (0.39 to 3.93)	NS NS NS NS NS	0.06
	*Toxocara*	Hookworm *Trichuris* Location Mainit Location San Isidro Location Trento	8.53×10^–5^ (4.27×10^–2^ to 1.71×10^1^) 2.04×10^–4^ (2.19×10^–8^ to 1.90) 1.83 (2.72×10^–3^ to 1.23×10^3^) 0.53 (4.78×10^–6^ to 5.97×10^4^) 8.63 (1.34×10^–2^ to 5.58×10^3^)	NS NS NS NS NS	0.05
**Cats**	*Trichuris*	Hookworm *Toxocara* Location Mainit Location San Isidro Location Trento	5.98×10^8^ (-) 0.46 (0.01 to 23.52) 7.14 (-) - 8.48×10^–8^ (-)	NS NS NS - NS	0.40
	Hookworm	*Trichuris* *Toxocara* Location Mainit Location San Isidro Location Trento	9.83×10^–7^ (-) 1.34 (0.15 to 12.11) 0.16 (0.007 to 3.47) - 1.55 (0.12 to 19.74)	NS NS NS - NS	0.23
	*Toxocara*	Hookworm *Trichuris* Location Mainit Location San Isidro Location Trento	1.33 (0.15 to 11.98) 0.73 (0.03 to 19.25) 2.76 (-) - 3.62×10^–8^	NS NS NS - NS	0.17
**Pigs**	*Ascaris*	*Trichuris* Strongyle Location Mainit Location San Isidro Location Trento	6.88 (2.40×10^–2^ to 1.97×10^3^) 2.08 (2.07 to 2.09) 3.19 (1.66×10^–2^ to 6.11×10^2^) 0.35 (0.35 to 0.36) 5.06 (4.22×10^–2^ to 6.08×10^2^)	NS 2×10^–16^*** NS 2×10^–16^*** NS	0.02
	*Trichuris*	*Ascaris* *Strongyle* *Location Mainit* *Location San Isidro* *Location Trento*	4.80 (1.44 to 15.94) 3.00 (0.96 to 9.40) 1.69 (0.16 to 18.90) 1.50 (0.13 to 17.06) 6.64 (0.73 to 60.67)	0.011* NS NS NS NS	0.32
	Strongyle	*Ascaris* *Trichuris* Location Mainit Location San Isidro Location Trento	2.16 (0.65 to 7.24) 2.79 (0.89 to 8.80) 1.49 (0.40 to 5.65) 5.89 (1.66 to 20.84) 4.91 (1.32 to 18.27)	NS NS NS 0.006** 0.018*	0.14

Abbreviation: NS, not significant. Asterisks (*) indicate significance.

In humans, a statistically significant positive association was found between *Ascaris* and *Trichuris* infection, and between *Ascaris* and hookworm infection (Table 2). Moreover, odds of infection in humans appear higher in Mainit and San Isidro compared with Bunawan (the reference location) for these parasites. The McFadden's R-squared suggests that the model fit to the data explains a moderate amount of the variation (13–21%) (Table 2). Additional results for human hosts without considering age are provided in Supplementary Table S1.

Across the three animal hosts analysed, the only significant association was found between *Trichuris* and strongyles in pigs (Table 2). We also found that the odds of strongyle infection in pigs was higher in San Isidro and Trento compared with Bunawan (the reference location). Even although no association was found between parasites in most animal hosts, the McFadden's R-squared was relatively high in some models, such as a 0.38 value found for *Trichuris* in cats. The age of the participants shows an association with *Ascaris* infection in humans but has not been shown to significantly alter the presence of *Trichuris* or hookworm infections.

**Figure 2.**
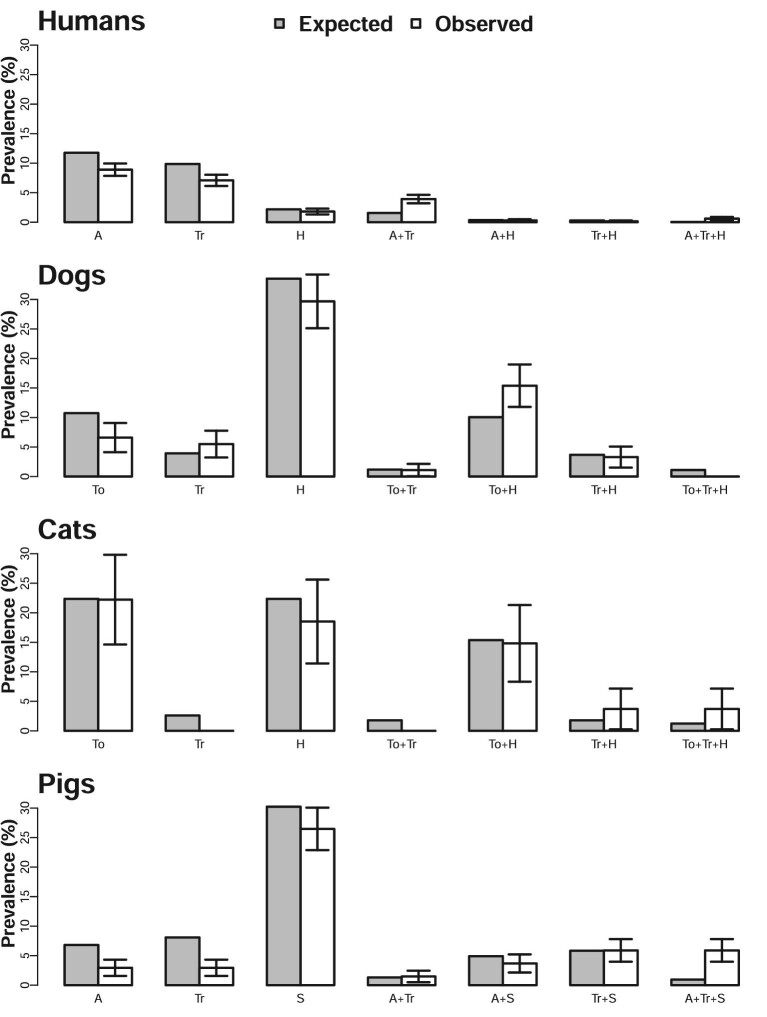
Observed and expected prevalence of single and co-infections. Bars on observed values are 95% CI. Values for no infections are summarised in Supplementary Table S2. A: *Ascaris*; H: hookworm; Tr: *Trichuris*; To: *Toxocara* and S: strongyles.

Co-infection with multiple species is consistent with random events when the expected prevalence is within the CI of the observed prevalence. This was generally the case across the four hosts, except in humans infected with *Ascaris* and *Trichuris*, dogs infected with *Toxocara* and hookworm and triple-infected pigs (*Ascaris, Trichuris* and strongyles), where higher co-infection prevalences than expected were observed (Figure 2). The observed and expected prevalence values of no infection are provided in Table S2.

## Discussion

We analysed data on intestinal helminth infections in humans and animals from four villages in the southern Philippines to determine associations between infections with different helminth species. In humans, the highest occurrence of *Ascaris* compared with other parasite species is in line with a previous study conducted by Silver et al.,^18^ which focuses on the geographical distribution of STHs in South Asia and South East Asia including the Philippines.

Positive associations were observed between *Ascaris* and *Trichuris* in humans and pigs, and *Ascaris* and hookworm in humans. The former is not surprising, as *Ascaris* and *Trichuris* share a faecal-oral transmission route. In addition, both have similar geographical distributions and are often prevalent in the same communities.^19^ This suggests that social and behavioural factors that lead to infection with one species are likely to increase the probability of infection with the other species. On the other hand, hookworm parasitic transmission is by the percutaneous route; hence, positive associations between hookworm and *Ascaris*, or between *Ascaris* and hookworm in humans, may potentially be due to increased predisposition to multiple infections in some individuals, or else due to similar exposures to parasite eggs or larvae contaminating the environment.^19^ There was no significant association found between the parasites examined in dogs and cats. This could be due to the limited number of samples, particularly in cats, with only 27 samples collected.

It is not surprising to find differences by location, in particular as Mainit and San Isidro are in a different province to Bunawan and Trento, with the former two in Surigao del Norte, while the latter two are in the Agusan del Sur province. Even although relatively close geographically and with broadly similar climatic conditions, San Isidro has a coastal location on an island, Mainit is near a large lake, whereas neither Bunawan nor Trento have a coastline or large water bodies nearby. This will affect the free-living stages of these parasites, which are influenced by several factors including micro-climatic suitability, sanitation and hygiene, and environmental contamination.^10^ The intensity of infection was found to be very different across sites and the three parasite species examined in humans (Supplementary Table S3).

Due to the higher than expected occurrence of co-infections between some parasites found here, our results suggest that factors such as climatic conditions, intestinal species-specific within-host interactions, host density levels, host behaviours and host physiological conditions, as well as other host-related factors or other environmental factors, are likely important determinants of the distribution of STH co-infections, rather than the parasites interacting synergistically.^10^ In other words, the co-infections may be due to common risk factors that can generate a purely statistical association among parasites.^10^

Environmental conditions could have an impact on host physiology and susceptibility to parasites. For example, a stressed or malnourished host is more likely to be infected. Host life history traits such as growth rate, lifespan and fecundity have also been shown to favour co-infection.^20^ Consequently, as suggested by our results, the distribution of multiple species infections may not occur independently, with associations known for some parasites, such as between *Ascaris* and *Trichuris*, presented in Howard et al.^21^ Thus, the presence of infection with one helminth may influence the outcome of infection with other helminth species.^22^

In the setting adopted in this study, dual and triple infections with different helminths were observed in humans, with a higher occurrence of dual infection of *Ascaris* and *Trichuris* than expected. In other studies, higher prevalences of co-infections were also found in humans, likely reflecting overall higher prevalences of helminth infections.^14^ Many co-infections were found in animals, some higher than expected, such as dual infections of *Toxocara* and hookworm in dogs. This could be due to higher levels of exposure to the environment compared with humans, as household animals in the Philippines are in many cases free roaming, eating all manner of possibly contaminated foods, which could cause co-infections.

Some studies have reported co-infections in animals such as cats and dogs, for instance, Pumidonming et al.^23^ reported dual co-infection of hookworm and *Toxocara* in dogs and cats with a prevalence of 1% and 0.6%, respectively. Here we found a co-infection prevalence of the same parasites of 15% and 14% for dogs and cats, respectively. Dual and triple co-infection were also observed by Symeonidou et al.,^24^ who studied pigs in Northern Greece, while we found higher than expected triple infections in pigs (Figure 2).

The dual or triple infections could have synergistic effects on the pathology of some infections.^8^ For instance, children co-infected with hookworm and *Trichuris* have been shown to be more likely to have blood haemoglobin levels indicative of anaemia than children harbouring only one of these parasites. Similarly, children with these dual infections who were treated were observed to have larger gains in blood haemoglobin concentrations over 12 mo compared with children who remained uninfected during the study.^25^ Furthermore, the immune response to a potential vaccine against one infection may influence response against another, and thus the effect of co-infections should be considered in vaccine development.^8^

Another interesting result from this study is that *Ascaris* seems to have a strong interaction with other helminths species, especially if they have a common mode of transmission (Table 2).^26,27^ This result is particularly interesting in pigs, since *Ascaris* is the least prevalent of the three species analysed (Figure 1).

Given that many intestinal helminth species present in the animal hosts are zoonotic and could cause disease in humans, it is important to consider possible transmission routes between the different hosts. Transmission from animals to humans could occur due to the consumption of drinking water contaminated with animal faeces or crops that have been fertilised using manure, with poor sanitation and proximity to infected animals being clear risk factors.^4,28^ The relatively high prevalence of intestinal helminth infections in dogs, cats and pigs that share an environment with the human populations reported in this study suggests zoonotic transmission.^2,4^ However, the importance of co-infection in processes of zoonoses emergence remains to be fully assessed. Accordingly, there is a need to focus more research in this area.

## Conclusion

The results obtained in this study showed positive associations between some, but not all, intestinal helminth species in humans and animals (pigs). Such associations were observed to be dependent on factors such as location and less dependent on the age or gender of the human population. Multiple infections (dual and triple) were predominantly observed in animals, with some infrequent, but higher than expected, dual infections in humans. The predisposition of the host, correlated exposures and interactions between species, with consideration of other environmental related factors (such as open defecation, sanitation and hygiene), could account for the multiple infections.

As co-infection could have an influence on the symptoms, duration and how infectious diseases respond to treatment, it is very important to further investigate the way co-infections influence parasite transmission in humans and animals to understand variations in infectious disease incidence and obtain appropriate diagnoses. In addition, it is necessary to account for the consequences of host exposure to multiple parasites to develop effective disease-prevention measures.

Overall, our study highlights that zoonotic transmission is very likely in these communities, and co-infections, which are present in a number of hosts, are not random and could point to synergistic interactions between parasites species. While more research is needed to quantify zoonotic transmission, particularly in the context of new emerging threats, it is clear that integrated control programmes and one health approaches that account for multiple hosts and target various parasites species are bound to be more successful in controlling disease in these populations.

## Supplementary Material

trac002_Supplemental_Tables

## Data Availability

Data is available upon request.
