# Supplementary material for: Co-infection of intestinal helminths in humans and animals in the Philippines
Source: Trans R Soc Trop Med Hyg. 2022 Feb 16;116(8):727–35. doi: 10.1093/trstmh/trac002 (PMC9356178; doi:10.1093/trstmh/trac002)
Supplement: trac002_Supplemental_Tables [file trac002_supplemental_tables.zip › Table S1.docx]

|  | Species | Variable | OR | p–value | McFadden’s R^2^ |
| --- | --- | --- | --- | --- | --- |
|  | Ascaris | Trichuris  Hookworm  Location Mainit  Location San Isidro  Location Trento | 4.1080  3.0841  3.6183  1.7106  0.6834 | 1.73 × 10^-6^ ***  0.04622 *  0.000431 ***  NS  NS | 0.118 |
|  | Trichuris | Ascaris  Hookworm  Location Mainit  Location San Isidro  Location Trento | 4.1264  0.9947  10.3923  27.4417  2.4748 | 1.72 × 10^-6^ ***  NS  0.00177 **  6.98 × 10^-6^ ***  NS | 0.203 |
|  | Hookworm | Ascaris  Trichuris  Location Mainit  Location San Isidro  Location Trento | 3.0825  1.0891  7.5251 × 10^-8^  6.2105  1.8077 | 0.0452 *  NS  NS  0.0205 *  NS | 0.168 |

**Table S1:** Multiple logistic regressions assessing associations between species in humans. Species variables are the presence or absence of infection. Age is not inclusive. Asterisk in p-value denote statistically significant results.

**Humans**

NS: not significant; Asterisks (*) indicates significant
