# Supplementary material for: Co-infection of intestinal helminths in humans and animals in the Philippines
Source: Trans R Soc Trop Med Hyg. 2022 Feb 16;116(8):727–35. doi: 10.1093/trstmh/trac002 (PMC9356178; doi:10.1093/trstmh/trac002)
Supplement: trac002_Supplemental_Tables [file trac002_supplemental_tables.zip › Table S2.docx]

Table S2 – Expected and observed values for no infections across the four hosts species.

| Host | Expected | Observed |
| --- | --- | --- |
| Humans | 0.74 | 0.77 (CI: 0.76-0.79) |
| Dogs | 0.36 | 0.38 (CI: 0.34-0.43) |
| Cats | 0.33 | 0.37 (CI: 0.28-0.46) |
| Pigs | 0.42 | 0.51 (CI: 0.47-0.55) |
