# Supplementary material for: Co-infection of intestinal helminths in humans and animals in the Philippines
Source: Trans R Soc Trop Med Hyg. 2022 Feb 16;116(8):727–35. doi: 10.1093/trstmh/trac002 (PMC9356178; doi:10.1093/trstmh/trac002)
Supplement: trac002_Supplemental_Tables [file trac002_supplemental_tables.zip › Table S3.docx]

| Location | *Ascaris* | *Trichuris* | *Hookworm* |
| --- | --- | --- | --- |
| Bunawan | 19.714286 | 0.14285714 | 0.01488095 |
| Trento | 1.841379 | 0.07586207 | 1.02758621 |
| Mainit | 117.541436 | 1.30939227 | 0 |
| San Isidro | 23.852071 | 1.40236686 | 0.18934911 |

Table S3 – Average number of eggs found in humans in the four different locations per parasite species. Note that EPG can be calculated by multiplying the number shown by 24.
